# Report on Successful Treatment of Refractory MAC 2 Lung Disease in Two Elderly Patients with Inhaled Liposomal Amikacin (ALIS) at Half the Standard Dose

**DOI:** 10.3390/diseases14020065

**Published:** 2026-02-11

**Authors:** Kenjiro Nagai, Syo Nagai

**Affiliations:** 1 Medical Corporation Ebino Centro Clinic, Ebino 8894304, Miyazaki Prefecture, Japan; centro.hp1998@gmail.com; 2 Respiratory Disease Center, Department of Medicine, Yokohama City University School of Medicine, Yokohama 2210855, Kanagawa Prefecture, Japan

**Keywords:** pulmonary MAC disease, liposomal amikacin (ALIS), nontuberculous mycobacteria (NTM), innovative drug delivery system, new drug delivery system

## Abstract

“Nontuberculous mycobacteria” (NTM) is a general term for mycobacteria other than the Mycobacterium tuberculosis complex and Mycobacterium leprae. In Japan, 90% of pulmonary NTM disease cases are caused by two species, Mycobacterium avium and M. intracellulare, which are collectively referred to as Mycobacterium avium complex (MAC) due to their biochemical similarity. Pulmonary MAC disease is broadly classified into fibrocavitary and nodular/bronchiectatic types, each of which exhibits distinctive pathological features. The pulmonary NTM disease incidence has been found to be 14.7 cases per 100,000 population per year, suggesting that Japan has the highest incidence of this disease in the world, and its incidence has also been shown to have already exceeded that of pulmonary tuberculosis. In addition, many elderly people have weakened immune systems, which often causes a decline in comprehension, and many medications for this have side effects, making it difficult to continue taking them and leading to treatment difficulties. The two cases reported here were both elderly women with refractory MAC lung disease, but they had different phenotypes: a fibrocavitary type and a long-standing, progressive nodular and bronchiectatic type. Treatment was performed with a regimen using Liposomal amikacin (ALIS), which is an aminoglycoside antibiotic that works by binding to bacterial Riposomes and inhibiting protein synthesis. Using amikacin Liposomal technology and a specialized inhaler, ALIS efficiently reaches alveolar macrophages, directly killing the MAC bacteria within. However, the unique administration method requires inhaler cleaning, making continued use difficult given the characteristics of patients with refractory MAC pulmonary disease. Even when treatment is possible, frequent side effects, such as hoarseness and dysphonia, while not severe, further contribute to the difficulty of initiating treatment. In both cases reported here, continued administration of rifampicin was difficult due to side effects such as liver damage and loss of appetite, and the patients’ conditions were also resistant to treatment, so ALIS was chosen, as it is thought to be more effective than other drugs and to have fewer systemic side effects. The patient had a limited understanding of how to clean the inhaler and how to inhale, making continued treatment difficult; therefore, we explained the efficacy and safety of ALIS to the patient’s family. Inhalation therapy is an effective method for delivering medication directly to the lungs, where the disease is located, while reducing systemic side effects. Until now, no inhalation therapy has existed for pulmonary MAC disease, and inhalation therapy itself is still a groundbreaking treatment administration method. This is the first case in the world where therapeutic efficacy has been confirmed with fewer than half the number of treatments required for standard treatment. Furthermore, as a new drug delivery method, inhalation offers a novel treatment option when existing medications are unavailable or ineffective for some reason, and it may be safe for use in elderly patients.

## 1. Background

“Nontuberculous mycobacteria” (NTM) is a collective term for mycobacteria other than the Mycobacterium tuberculosis complex and Mycobacterium leprae. NTM includes over 150 species, which are widely distributed not only in natural environments such as water and soil but also in residential environments such as bathrooms. Inhalation exposure from these environments can lead to respiratory infection and pulmonary NTM disease. In Japan, 90% of cases of this disease are caused by two species, Mycobacterium avium and M. intracellulare [1], which are collectively referred to as Mycobacterium avium complex (MAC) because the two species share similar biochemical profiles. Pulmonary MAC disease is broadly divided into the fibrocavitary and the nodular–bronchiectatic types [2,3,4], each with its own distinctive features [5,6] (Table 1).

The course and prognosis of pulmonary MAC disease vary depending on the type. The fibrocavitary type generally progresses rapidly, and chemotherapy is recommended early after diagnosis. Because the nodular/bronchiectatic type progresses slowly and varies widely from case to case, there is no clear basis for determining when to start chemotherapy after diagnosis, and it is generally left to the clinician’s discretion.

The pulmonary NTM disease incidence has been found to be 14.7 cases per 100,000 people per year [2], suggesting that Japan has the highest incidence of this disease in the world and that its incidence has already exceeded that of pulmonary tuberculosis. As incidence increases with age, this case report will be important for the future treatment of pulmonary MAC disease [2].

Diagnostic methods consist of clinical and bacteriological criteria, and a diagnosis is made when both are met. The former includes the ability to exclude other diseases based on chest imaging findings, while the latter includes positive cultures of at least two different sputum samples. It is clearly stated that positive cultures cannot be substituted for positive nucleic acid amplification tests, and that gastric juice samples have little diagnostic value.

In Japan, where chemotherapy is primarily based on three drugs—clarithromycin (CAM), ethambutol (EB), and rifampicin (RFP)—it is recommended to administer these three drugs orally on a daily basis, with intramuscular streptomycin or kanamycin added as needed [7,8,9]. Treatment methods are shown in Table 2.

**Figure 1:**
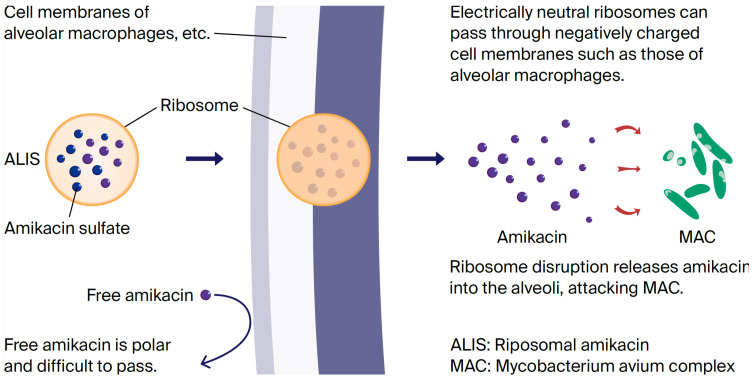
ALIS mechanism of action [13].

Regarding the drug administration duration, although one guideline stipulates that the duration of drug administration should be “approximately one year after bacterial culture negativity,” there is little evidence, and this is a topic for future research. Amikacin was originally marketed as an intravenous formulation and approved for the treatment of fibrocavitary pulmonary edema and refractory nodular/bronchial pulmonary edema. However, its therapeutic efficacy remains unclear. With standard drug therapy, the long-term bacterial response rate remains approximately 60% [10], with a meta-analysis reporting a rate as low as 40% [11]. A new drug, liposomal amikacin (ALIS), is an aminoglycoside antibiotic that acts by binding to bacterial liposomes and inhibiting protein synthesis. A comparison of the lung and plasma AUCs of amikacin administered via various administration methods in rats showed that the lung AUC was more than 1800-fold higher with liposomal administration than with plasma, demonstrating a greater difference between the lung and plasma levels compared with intravenous administration and inhaled amikacin. For ALIS to reach the alveoli in the periphery of the lung and effectively attack MAC, it is important that it be taken up by macrophages, the primary cells infected by MAC. ALIS’s liposomal lipids are non-polar and easily pass through negatively charged cell membranes, making them more easily taken up by macrophages than non-polar amikacin. Its greatest features are its liposomal amikacin technology and dedicated inhaler. This allows amikacin to efficiently reach alveolar macrophages and directly act on MAC bacteria present therein, killing them. A diagram is provided to help understand this mechanism of action (Figure 1). This makes it an innovative treatment that exerts therapeutic effects while minimizing systemic side effects [12,13,14].

Inhaled amikacin (ALIS) is indicated for fibrous cavitary stenosis and other refractory cases. However, although there is information on its efficacy and side effects in several domestic case reports (described later), due to its high cost and difficult inhalation method, there is still a lack of data on its therapeutic efficacy in actual clinical practice. Here, we report two cases: one in which a cancer patient with weakened immune function developed severe pulmonary MAC disease, and the other in which the disease gradually progressed over several years. Both cases involved elderly women, and ALIS was effective at lower doses compared to the standard therapeutic dose.

## 2. Case Presentation and Outcome

### 2.1. Case 1: 73-Year-Old Female

Chief complaint: Dyspnea and cough.

Medical history: Stage I breast cancer.

Lifestyle history: Smoking history: none. Alcohol consumption: 1 beer/day.

**Figure 2:**
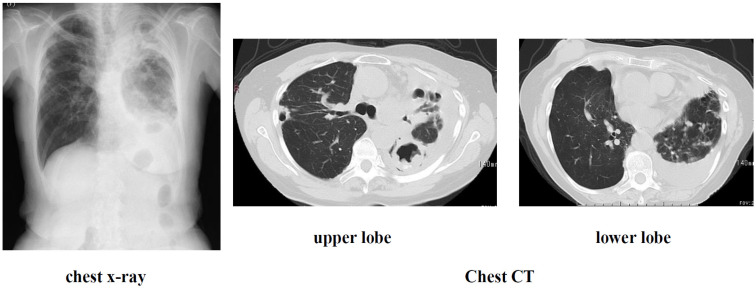
Imaging findings at admission.

Present illness: The patient had been diagnosed with left pleural effusion and pulmonary MAC disease and was receiving treatment at a local hospital. She was referred to our hospital due to worsening dyspnea and chronic cough. Right breast cancer was incidentally discovered during the examination, so she was referred to a breast cancer specialty hospital. Due to the early stage of the disease, a right mastectomy was performed, but given her advanced age and impaired reading ability due to frailty, postoperative chemotherapy was not administered. Regarding pulmonary MAC disease, chest CT scan revealed diffuse granular opacities in both lung fields, a cavity in the left upper lobe, and pleural effusion in the left pleural cavity (Figure 2). Sputum acid-fast bacillus cultures detected M. intracellulare twice; furthermore, blood tests were positive for MAC antibodies, and a diagnosis of pulmonary MAC disease was made. Thoracentesis and pleural fluid cytology revealed no cancer, and bacterial cultures did not detect any bacteria, so a diagnosis of pulmonary MAC disease with pleurisy was made. Outpatient treatment of 800 mg/day CAM, 300 mg/day RFP, and 250 mg/day EB was initiated, but symptoms did not improve, so the patient was admitted to the hospital for ALIS introduction.

Characteristics at the time of admission included a height of 145 cm, a weight of 35 kg, a temperature of 36.5 °C, a blood pressure of 124/78 mmHg, a pulse rate of 98/min, and an irregular pulse.

Others included no palpable lymph nodes, cardiac rhythm irregularities, lung sounds with coarse and intermittent rales, and a respiratory rate of 21/min, and the abdomen was flat, soft, and nontender. A right mastectomy was performed, and no limb abnormalities were observed.

**Figure 3:**
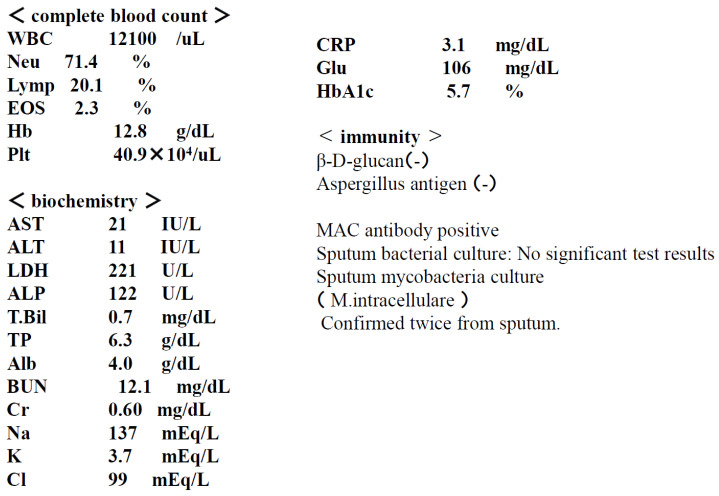
Test results at the time of admission. MAC antibody detects IgA antibodies against the cell wall component (GPL core) of MAC bacteria.

Examination findings at the time of admission are shown in Figure 2 and Figure 3.

**Figure 4:**
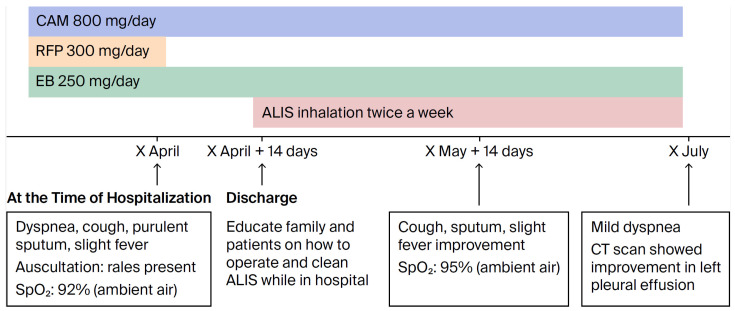
Progress after hospitalization. CAM: clarithromycin; RFP: rifampin; EB: ebutor; ALIS: amikacin.

Post-discharge course: RFP was discontinued due to liver dysfunction and anorexia onset upon admission. CAM and EB therapy were continued, and fluid replacement therapy was administered while awaiting improvement in the patient’s anorexia and liver damage. Approximately 14 days were then spent instructing the patient’s family on how to operate and clean the inhaler before starting ALIS. After the patient’s anorexia and liver damage improved and she was able to inhale properly, she was discharged and began treatment with ALIS administered once daily and then twice weekly. The change from daily to twice-weekly administration was due to the patient’s poor general condition, which made it difficult for her to consistently administer the inhaler herself; therefore, administration was initiated on days when her family was available to assist (Figure 4).

**Figure 5:**
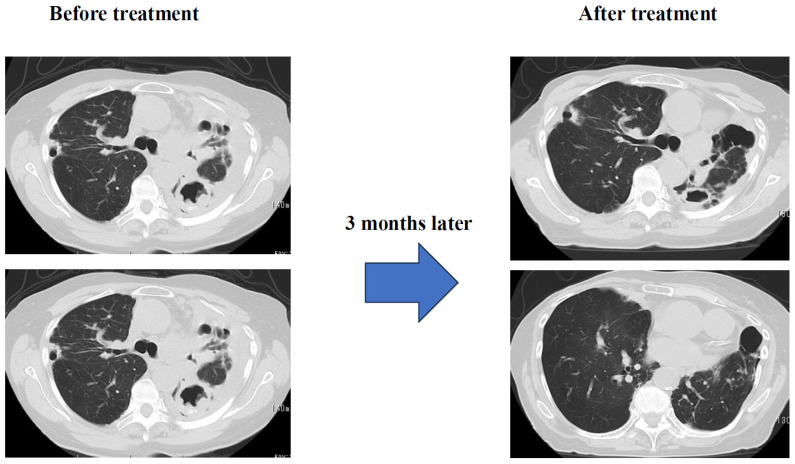
Image of progress before and after treatment.

Results: Three months later, sputum testing detected M. intracellulare, but chest CT scans showed significant improvement in pleural effusion and infiltrates (Figure 5). Subjective symptoms included almost complete resolution of coughing and significant improvement in dyspnea. SpO_2_ (outside air), which was 92% at the start of treatment, improved to approximately 97%. Currently, the patient is receiving CAM, EB, and ALIS in one inhalation once a day, twice a week, and her condition is stable.

### 2.2. Case 2: 87-Year-Old Female

Chief complaint: Dyspnea and cough.

Past medical history: Schizophrenia.

Lifestyle history: Smoking history: none. Alcohol consumption: occasional.

**Figure 6:**
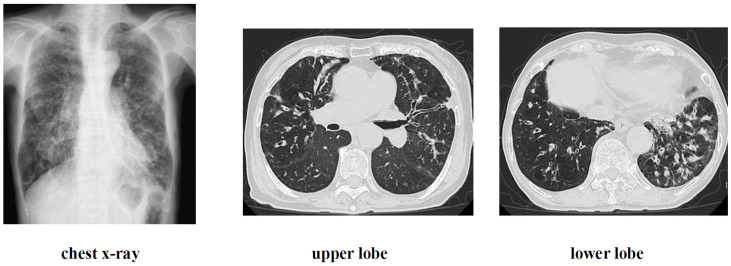
Imaging findings at admission.

**Figure 7:**
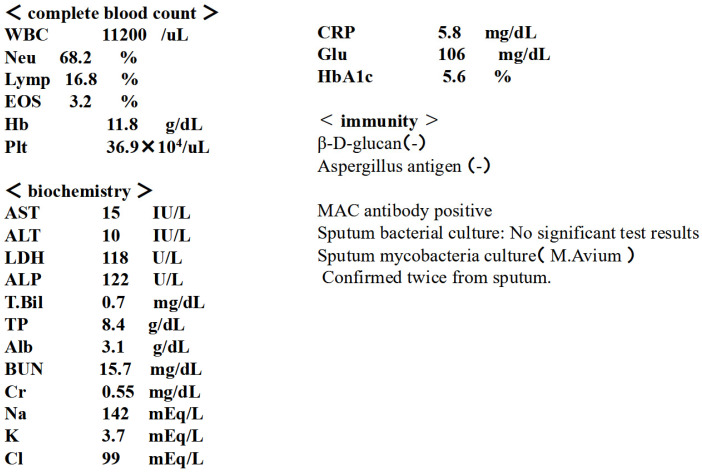
Test results at the time of admission. MAC antibody detects IgA antibodies against the cell wall component (GPL core) of MAC bacteria.

History of current illness: Approximately 3 years ago, the patient visited a local physician complaining of dyspnea, cough, and slight fever. Chest CT scan revealed granular and nodular shadows in both lung fields (Figure 6), while sputum culture revealed no significant bacteriological changes in the sputum. A blood test revealed positive MAC antibodies, leading to a tentative diagnosis of pulmonary MAC disease (Figure 7). Erythromycin (EM) was administered at 400 mg/day, but symptoms gradually worsened over approximately 3 years, with M. avium detected twice in sputum cultures. A nodular bronchiectasis diagnosis was made, and outpatient treatment with 800 mg/day CAM, 300 mg/day RFP, and 250 mg/day EB was initiated. Treatment continued for approximately one month, but the cough, low-grade fever, and dyspnea did not improve. Furthermore, imaging findings worsened, loss of appetite developed, and the patient’s overall condition worsened. Consequently, the patient was diagnosed with refractory pulmonary MAC disease and hospitalized, and ALIS therapy was initiated. At the time of admission, the patient’s height was 148 cm, weight was 32 kg, temperature was 37.5 °C, blood pressure was 98/78 mmHg, pulse rate was 98 beats/min, and arrhythmia was present.

**Figure 8:**
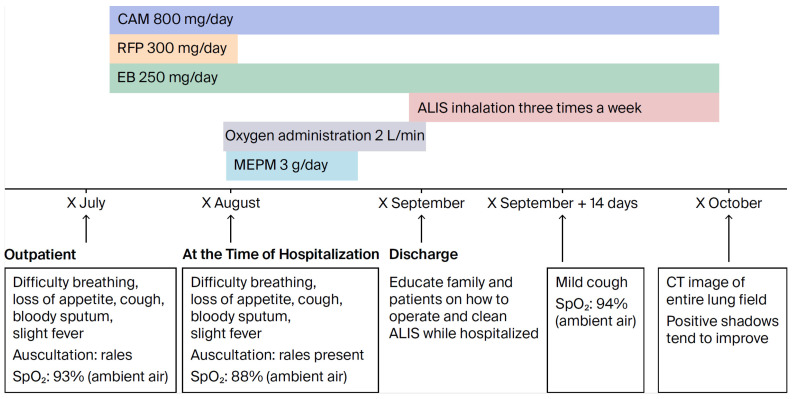
Progress after hospitalization.

No lymph nodes were palpable, cardiac rhythm was irregular, lung sounds were intermittent, respiratory rate was 19 breaths/min, and the abdomen was flat and soft, with no tenderness. A right mastectomy was performed, but no abnormalities were found in the extremities. Chest X-ray examination at the time of admission suggested possible bacterial pneumonia, and 3 g/day MEPM and fluid replacement were initiated to treat the patient’s appetite loss. Fever and dyspnea improved within approximately one week, so MEPM and oxygen administration were discontinued. During hospitalization, the patient and family received approximately two weeks of instruction on how to use and clean the ALIS inhaler. After discharge, treatment with ALIS was initiated with inhalation once daily, three times a week. This was because the patient’s advanced age and schizophrenia made it impossible to continue treatment without the presence of family members (Figure 8).

**Figure 9:**
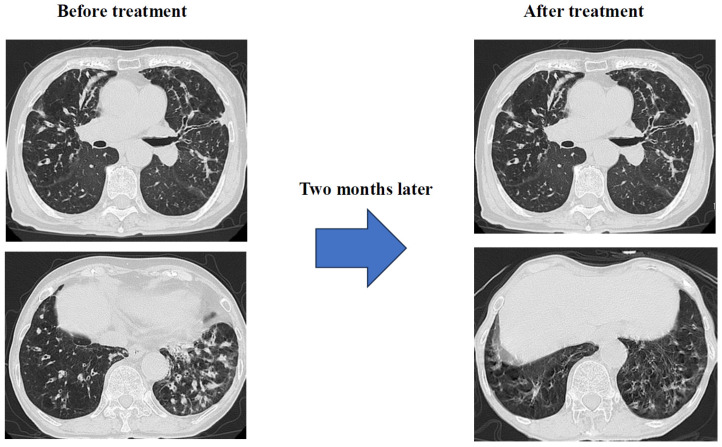
Image of progress before and after treatment.

Results: Just two months after starting treatment, dyspnea, cough, and low-grade fever had almost completely resolved. Chest CT scan showed significant improvement in granular and infiltrative opacities, particularly in the lower lobes (Figure 9).

Sputum acid-fast bacillus testing revealed M. avium, and treatment is ongoing with 800 mg/day CAM, 250 mg/day EB, and ALIS (single inhalation, once daily, three times weekly).

## 3. Discussion

In both cases reported here, rifampicin was difficult to administer due to its side effects, and the patients’ conditions were resistant to treatment; therefore, ALIS was selected as a treatment with potential for greater efficacy and fewer systemic side effects compared to other medications. Inhalation therapy is an effective method for delivering medication directly to the lungs—the disease site—and reducing systemic side effects. To date, no inhalation therapy has been available for pulmonary MAC disease, making this therapy itself a groundbreaking treatment method. These are the first cases in the world in which therapeutic efficacy has been confirmed, with fewer than half the number of treatments required for standard treatment.

**Figure 10:**
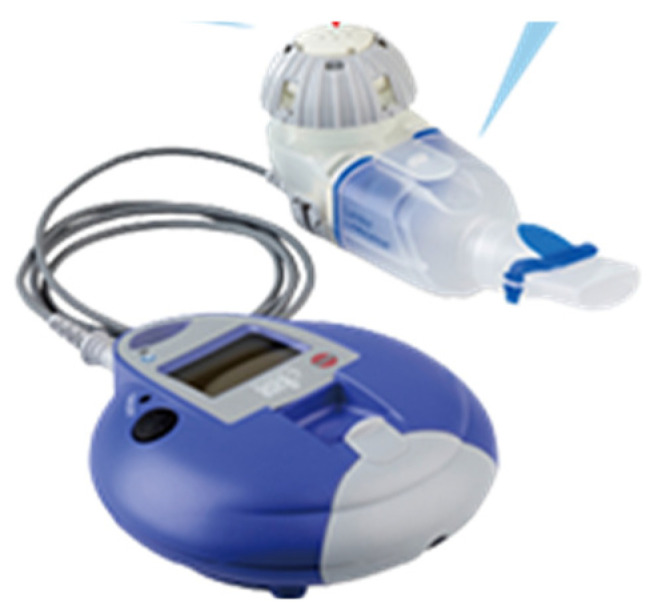
The ALIS inhaler [14].

Case 1 had a history of breast cancer, and it was thought that the immunosuppressive state caused by the cancer contributed to pulmonary MAC disease progression and led to a deterioration in the patient’s general condition. The diagnosis was fibrocavitary pulmonary MAC disease and pleurisy, the latter of which is considered rare in nontuberculous mycobacterial infection (NTM), but Ichiki et al. reported 9 cases among 309 cases of NTM disease, and left pleural effusion, as in this case, is common [15]. Thoracentesis and bacterial cultures did not identify any significant bacteria, but cytology revealed no malignant findings, consistent with a diagnosis of MAC pulmonary disease and pleurisy [16]. Initially, in accordance with the guidelines of the Japanese Respiratory Society and the Japanese Society for Tuberculosis, the patient was administered CAM 800 mg/day, RFP 300 mg/day, and EB 250 mg/day. However, due to severe liver damage and anorexia, likely due to RFP, continuation of treatment was difficult, and no improvement was observed. While intravenous amikacin is typically used, we decided to use ALIS, which is highly effective and has few side effects. ALIS is an aminoglycoside antibiotic that works by binding to bacterial ribosomes and inhibiting protein synthesis. The greatest feature of this innovative drug is its unique liposomal amikacin technology and dedicated inhaler (Figure 10), which efficiently delivers amikacin to alveolar macrophages and directly kills MAC bacteria [17]. Its efficacy has also been reported in Japanese patients, with mild side effects such as hoarseness [18,19]. Oshita et al. reported 11 cases in which an ALIS-based regimen was effective, but side effects such as hoarseness and dysphonia were unavoidable, making it difficult to continue treatment [20]. The treatment regimen was changed to CAM 800 mg/day, EB 250 mg/day, and ALIS 1 inhalation once daily, administered twice weekly. The reasons for changing to twice-weekly administration were the patient’s poor general condition, advanced age, and poor comprehension. Because the inhalation method and inhaler cleaning procedures were complicated and the patient was unable to administer ALIS independently, family supervision was deemed necessary, and twice-weekly administration was the most feasible administration frequency. Although the inhaled dose was less than half the usual daily dose, pleural effusion and imaging findings improved, and subjective symptoms also significantly improved, with no side effects observed. Case 2 was a patient strongly positive for anti-MAC antibodies on imaging and blood tests 3 years earlier, but a definitive diagnosis could not be reached due to a negative sputum culture for acid-fast bacilli. Although the patient received EM therapy, her symptoms worsened, likely due to a weakened immune system associated with aging. Sputum cultures for acid-fast bacilli tested positive for M. avium twice, confirming a definitive diagnosis of nodular and bronchiectatic pulmonary edema. Administration of 800 mg/day CAM, 300 mg/day RFP, and 250 mg/day EB was initiated, but symptoms such as fever, cough, and dyspnea, as well as imaging findings, showed little improvement. Appetite loss also developed, and her general condition worsened, with the former symptom thought to be due to the effects of RFP, so treatment was discontinued. Due to the intractable condition, the regimen was changed to 800 mg/day CAM, 250 mg/day EB, and one inhalation of ALIS once daily, three times weekly. The reason for administering ALIS three times weekly was that the patient was elderly and had schizophrenia, making it difficult for the patient to understand, and the family was only able to be present three days a week to monitor the administration. In this case, significant improvements in symptoms and chest imaging were observed just two months after starting treatment, with no side effects observed. Both patients were elderly women who were immunosuppressed due to comorbidities and aging and had developed severe MAC pulmonary disease; however, treatment was successful with an ALIS-based regimen.

## 4. Conclusions

ALIS is an aminoglycoside antibiotic that works by binding to bacterial liposomes and inhibiting protein synthesis. Its greatest feature is its groundbreaking and pioneering effect: by using amikacin Liposome technology and a dedicated inhaler, it efficiently reaches alveolar macrophages and directly kills the MAC bacteria within them, making it a novel drug delivery method. However, the unique administration method requires inhaler cleaning, and patients with refractory pulmonary MAC disease are often elderly, meaning that their cognitive decline often makes continued use difficult. However, it is more effective than other oral and intravenous medication options, and side effects are minor, such as hoarseness.

In these two cases, treatment was possible with the cooperation of the patient’s family, specifically with inhaler cleaning and medication inhalation assistance. No side effects were observed, and effective treatment was possible with fewer treatment sessions than usual. These results suggest that, as a new drug delivery method, inhaled antibiotics may not only provide a new treatment option when existing drugs are ineffective or cannot be used for some reason, but may also demonstrate high efficacy and safety, even in elderly patients. Furthermore, if the drug is effective with fewer inhalations, it may be possible to lower the barrier to introducing the drug.

## Figures and Tables

**Table 1:** Two major types of pulmonary MAC disease [4].

	Fiber Cavity Type	Nodular/Bronchial Dilatation Type
Typical patient profile	Middle-aged and elderly men with underlying diseases such as COPD and pneumoconiosis and a history of smoking	Non-smoking middle-aged and elderly women with no underlying diseases
Percentage of patients with pulmonary MAC disease	Declining trend	Increasing trend (more than 90% of newly diagnosed pulmonary MAC disease patients)
Chest X-ray image	Cavity and infiltrative shadows in the center of the upper lung field	Small nodular and linear shadows in the middle and lower lung fields
Uniformity of clinical course	Most cases have a similar course	Not uniform, but varies depending on the case
Outcome	Progresses relatively quickly, within 1 to 2 years	Progression over months or years (although some cases remain stable for long periods)
When to start chemotherapy	Chemotherapy should be initiated promptly after diagnosis, taking into consideration surgical options	Treatment should not be initiated immediately after diagnosis, but should be determined comprehensively based on the progression of the disease

**Table 2:** Chest computed tomography findings before and after the first treatment [7].

Disease Type	Treatment Regimen	
Non-cavitating nodular/bronchial dilatation type (excluding severe cases)	Use either Method A or Method B	
	Method A: Daily administration CAM 800 mg or AZM 250 mg EB 10~15 mg/kg (MAX 750 mg) * RFP 10 mg/kg (MAX 600 mg)	Method B: Administered 3 days a week CAM 1000 mg or AZM 500 mg EB 20~25 mg/kg (MAX 1000 mg) * RFP (600 mg)
Fiber cavity type Cavitated nodular/bronchial dilatation type Severe nodular and bronchiectatic type	Method A + initial treatment (3–6 months): combine with the following: SM 15 mg/kg or less (up to 1000 mg) 2–3 times a week by intramuscular injection or AMK 15 mg/kg daily or 15–25 mg/kg 3 times a week by intravenous infusion, adjusted with TDM (for those 50 years of age or older, 8–10 mg/kg 2–3 times a week, up to a maximum of 500 mg, adjusted with TDM) Consider combining with surgical treatment if necessary	
Refractory cases	Combine with Method A with one of the following: ALIS 590 mg/day inhalation or SM 15 mg/kg or less (up to 1000 mg) intramuscular injection 2–3 times per week or AMK 15 mg/kg daily or 15–25 mg/kg 3 times per week by intravenous infusion, adjusted with TDM (for those 50 years of age or older, 8–10 mg/kg 2–3 times per week, up to a maximum of 500 mg, adjusted with TDM) Consider combining with surgical treatment if necessary	

* means that it is not necessary to take it orally. AZM: Azithromycin; AMK: Amikacin; SM: streptomycin; TDM: Therapeutic Drug Monitoring.

## Data Availability

Data are available from the corresponding author upon reasonable request.
